# Preliminary investigation demonstrating the GHITM gene probably involved in apoptosis and growth of the golden apple snail (*Pomacea canaliculata*)

**DOI:** 10.1186/s12864-019-6434-2

**Published:** 2020-01-06

**Authors:** Wenchao Yu, Baolu Zhang, Hongce Song, Rui Zhan, Lingling Li, Cheng He, Qiuyun Jiang, Xiaona Wang, Lei Wei, Nannan Zhao, Wen Guo, Xiaotong Wang

**Affiliations:** 1 School of Agriculture, Ludong University, Yantai, 264025 China; 2 Oceanic Consultation Center, Ministry of Natural Resources of the People’s Republic of China, Beijing, 100071 China; 3 Key Laboratory of Aquaculture Nutrition and Feed (Ministry of Agriculture), Key Laboratory of Mariculture (Ministry of Education), Ocean University of China, 5Yushan Road, Qingdao, 266003 Shandong China; 4 Center for Mollusc Study and Development, Marine Biology Institute of Shandong Province, Qingdao, 266104 China

**Keywords:** *Pomacea canaliculate*, GHITM gene, Complete cDNA, RNAi, Cell apoptosis, Growth

## Abstract

**Background:**

Growth hormone inducible transmembrane protein (GHITM) is a highly conserved transmembrane protein. This study was conducted to investigate the role of GHITM gene in the apoptosis and growth of the golden apple snail Pomacea canaliculate.

**Results:**

The complete cDNA of this gene was cloned using the rapid amplification of cDNA ends (RACE) method and subjected to bioinformatics analysis. The full-length cDNA was 2242 bp, including an open reading frame of 1021 bp that encoded a protein of 342 amino acid residues. The mRNA expression profiles of GHITM gene in different tissues (liver, kidney, gonad and foot) and different growth phases (6-months old and 2-years old) showed that it was expressed in various tissues and different growth phases. Silencing of the GHITM gene by RNAi (RNA interference) experiments revealed that the GHITM gene possibly plays a role in inhibiting apoptosis through detecting the Caspase (Cysteine-requiring Aspartate Protease)-3 activity. In addition, the aperture width and body whorl length of the snail was significantly affected by RNAi, suggesting that this gene plays a significant role in promoting the growth of the organism.

**Conclusions:**

These results demonstrated that the GHITM gene was involved in apoptosis and growth in golden apple snail.

## Background

Growth hormone inducible transmembrane protein (GHITM) is a highly conserved transmembrane protein that exists widely in plants and animals [1]. This gene plays an important role in the growth and anti-aging mechanisms of the body. Reimers analysis revealed that there was a special structure (UPF005) at the C-terminus that is unique to the BAX inhibitor (BI)-1 superfamily [2]. Therefore, the GHITM gene was classified as a member of the BI-1 superfamily. Other members of this superfamily, responsive to centrifugal force and shear stress gene 1, Golgi anti-apoptotic protein and Lifeguard, all play roles in the growth and development of the body and inhibit apoptosis [3–6]. We speculate that GHITM also has similar functions, but the specific effects need further research and certification.

**Fig. 1:**
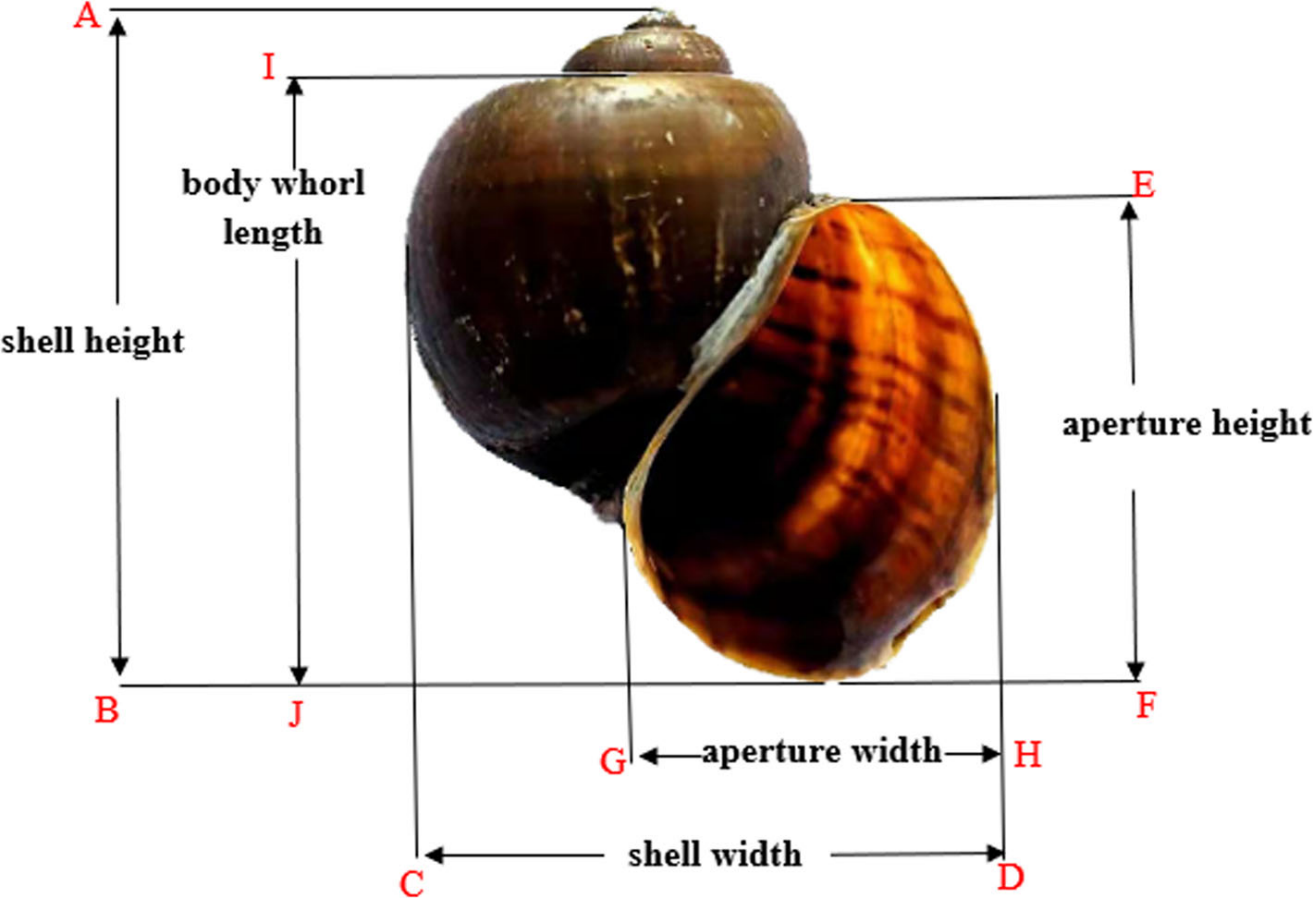
The measurement standards of the growth indexes. AB, shell height; CD, shell width;EF, aperture height; GH, aperture width; IJ, body whorl length

The golden apple snail (its body size traits were shown in Fig. 1.) originated in South America and was introduced into Asia as food in the 1980s [7, 8]. It has been listed as an invasive species owing to its characteristics of feeding on a variety of crops and rapidly reproducing. It also has a low economic value [9, 10] and serious damage to the development of the rice planting industry. In addition to the lack of natural enemies, its strong adaptability to temperature [11–15], food [16, 17] and wet environments [18], as well as its strong ability to grow and reproduce have been keys to its invasiveness. Studies of other species have shown that the GHITM gene functions in regulating the growth of the body and in cell apoptosis. Therefore, improving our knowledge of the GHITM gene of the golden apple snail may elucidate the mechanisms behind the golden apple snail’s strong adaptability and rapid growth and reproduction. In addition, golden apple snails are rich in fat and protein [19]; therefore, studying genes related to their growth may help increase their protein levels, making them feed for some economically important fish [20, 21]. The GHITM gene was first discovered in the brown fat of mice [1], and then was studied in *Crassostrea gigas* [22], *Anthocidaris crassispina* [23], *Bombyx mori* [24], *Actiniaria*, *Pinctada martensii* and *Apostichopus japonicus* [25]. To date, no studies on the GHITM gene in *Pomacea canaliculata* have been published.

Apoptosis is an important mechanism for the preservation of a healthy and balanced immune system in vertebrates [26]. For molluscs, more and more researches showed that apoptosis was related with maintenance of tissue homeostasis, responses to environmental stress, and fights against diseases [27]. Especially, hemocytes can participate in all steps of the immune response pathway as cellular mediators in molluscs [28], and integrins regulate the phagocytic ability of molluscan hemocytes and induce hemocytic apoptosis [29, 30].

In this study, the full-length cDNA sequence of the GHITM gene was cloned successfully, and the structure, properties and subcellular localization of the putative protein were predicted using bioinformatics software. The expression levels of the GHITM gene in different tissues of golden apple snail were assessed by quantitative real-time PCR. After RNAi-mediated knockdown for this gene, the growth and apoptotic indexes of the snail were evaluated and compared. The findings in this study will provide insights into the function of GHITM gene in the golden apple snail.

## Results

### Characterization of the GHITM cDNA sequence

**Fig. 2:**
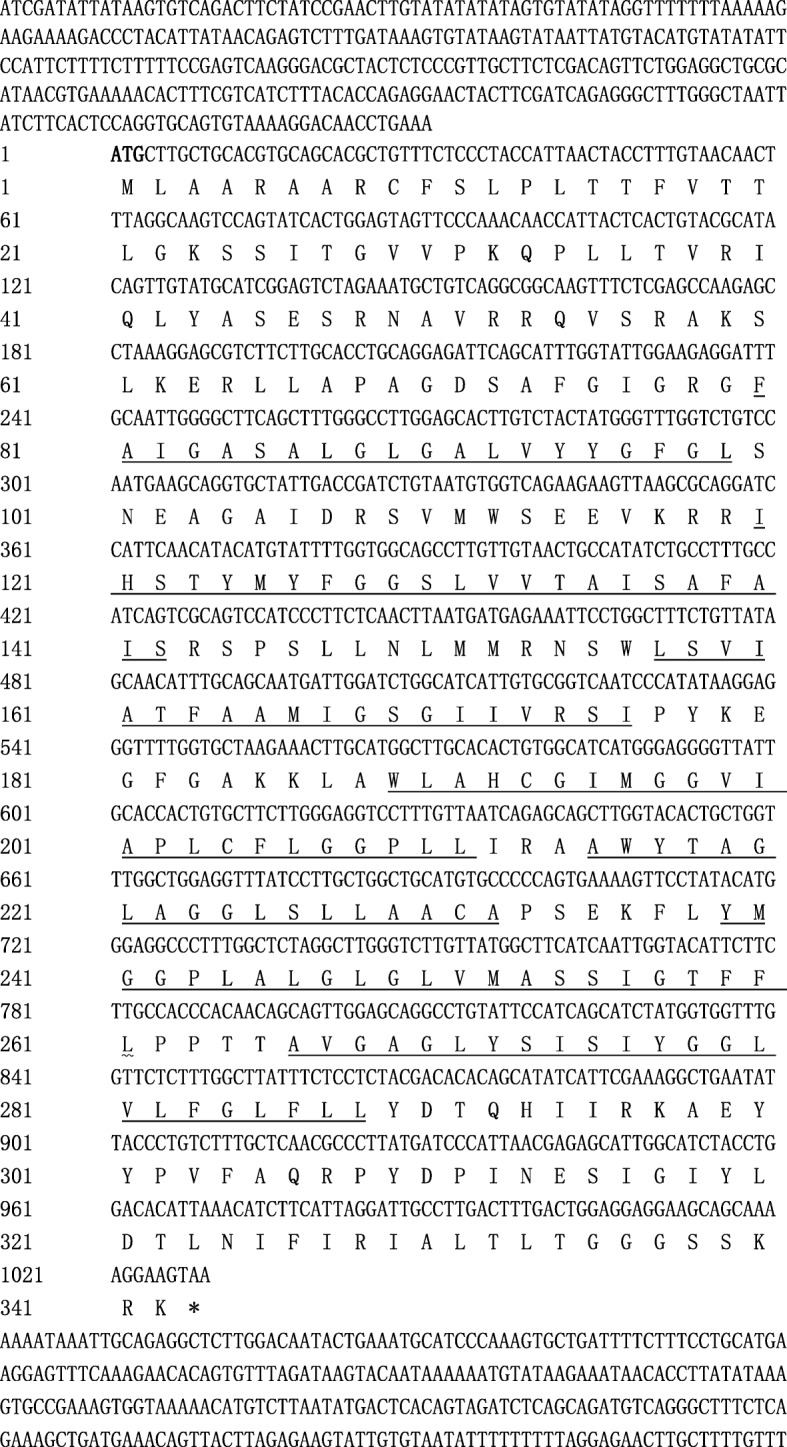
Nucleotide and deduced amino acid sequences of GHITM cDNA. The start code ATG is in bold, and the stop codon is indicated with an asterisk. In the 3′ UTR, the consensus polyA signal AATTAAA is double underlined. The amino acids of the transmembrane region are underlined

The full-length cDNA sequence of GHITM included a 5′- UTR (Untranslated Region) of 324 bp, an open reading frame of 1029 bp, encoding a protein of 342 amino acids, and a 3′-UTR of 890 bp. The GenBank accession number for this nucleotide sequence is MN219409. The characterization is shown as (Fig. 2).

### Bioinformatics analysis of GHITM

**Fig. 3:**
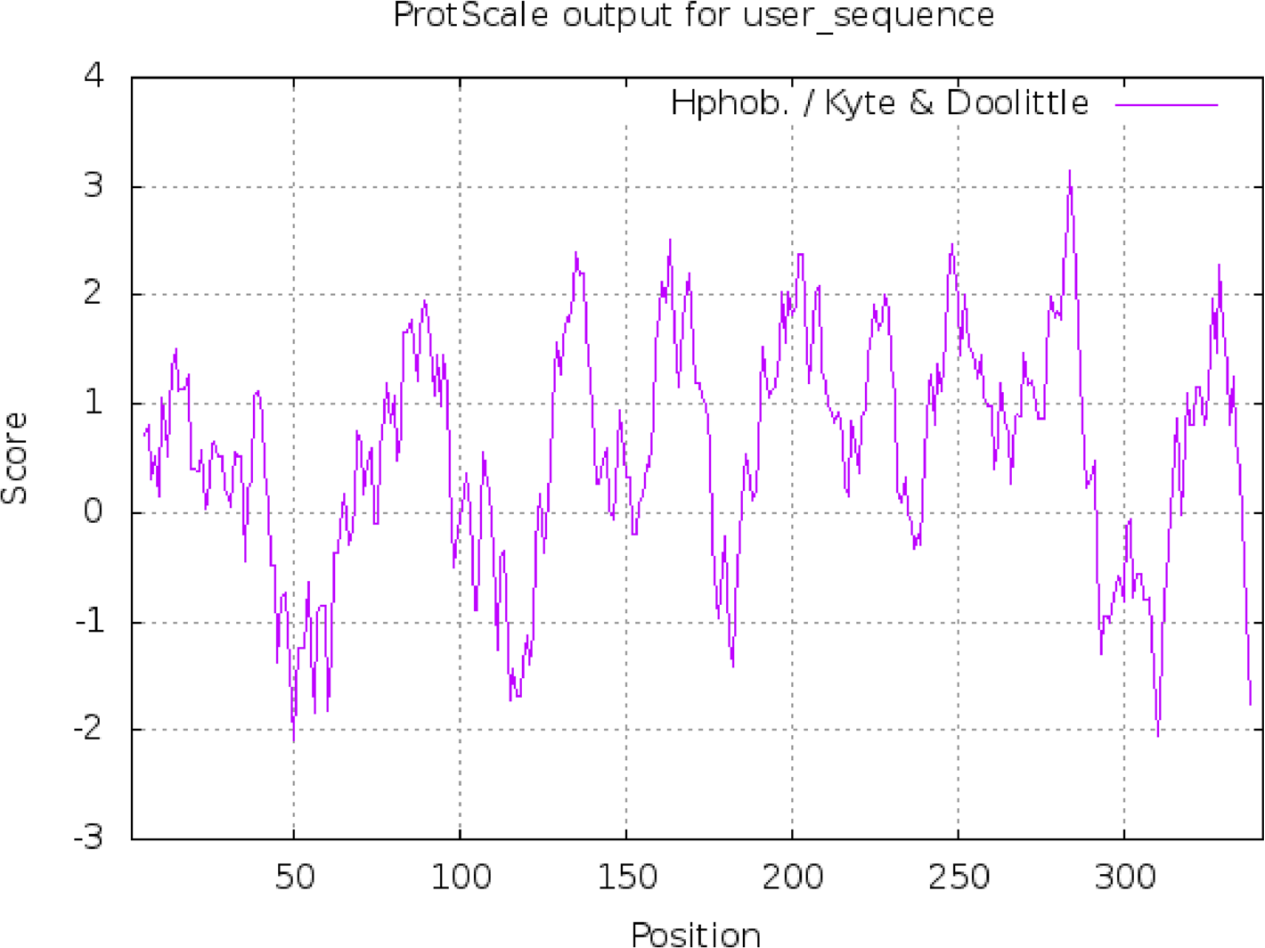
Hydrophilic and hydrophobic profile of the GHITM protein. Notes: The horizontal axis indicates the positions of amino acids in this protein and vertical axis indicates the hydrophobicity

**Fig. 4:**
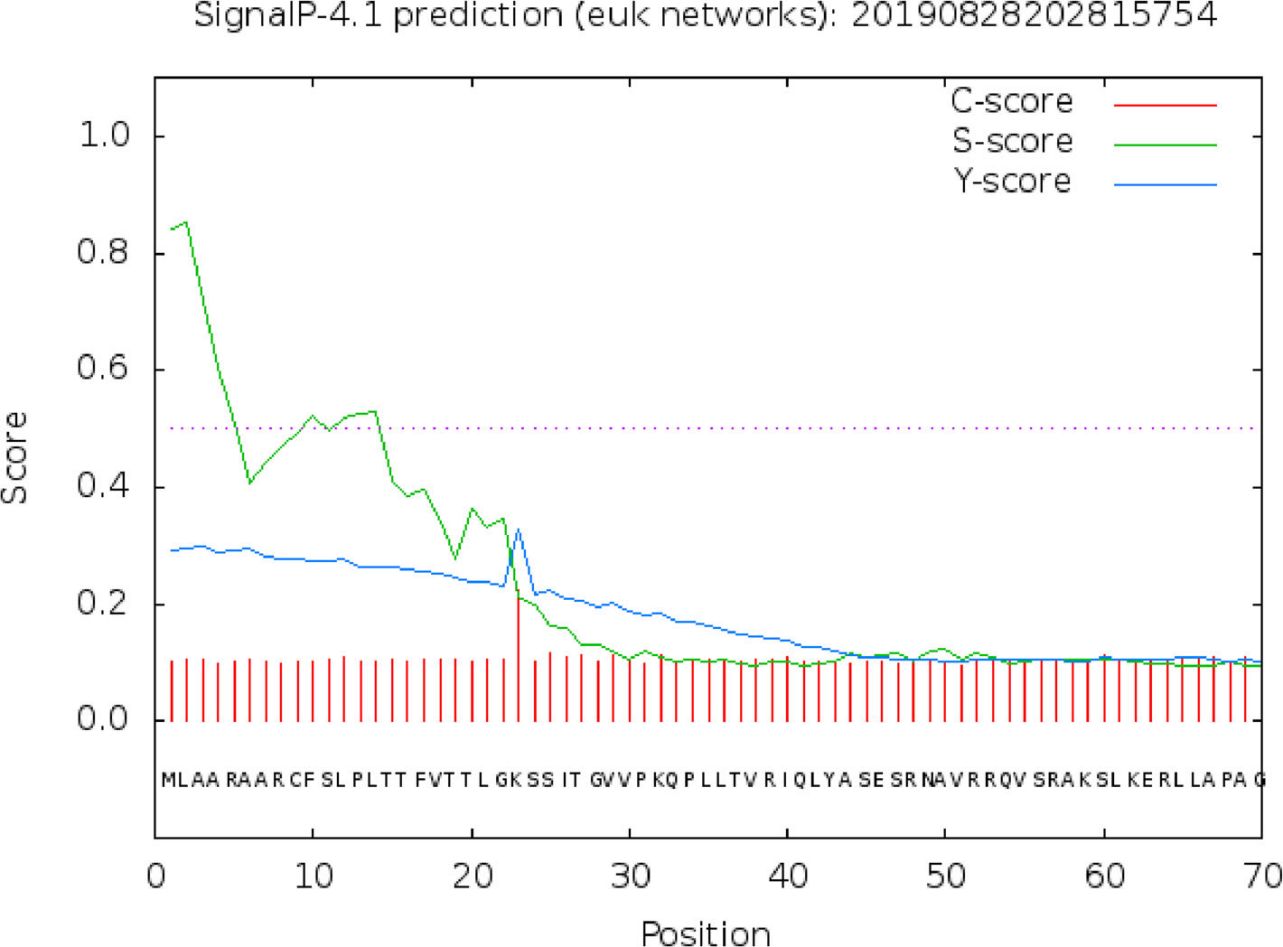
Prediction of the golden apple snail GHITM protein’s signal peptide. Note: C-score, raw cleavage site score. S-score, signal peptide score. Y-score, combined cleavage site score.

**Fig. 5:**
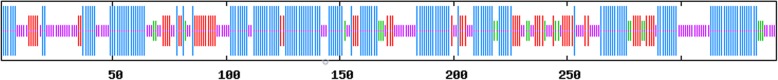
Prediction of the second structure of GHITM protein. Blue, red, green and purple stand for alpha helix, extended strand, beta turn and random coil, respectively

The identified open reading frame of GHITM encoded a putative protein of 342 amino acids with a predicted molecular mass of 36,373.87 Da and theoretical isoelectric point of 9.98. The total number of negatively charged residues (Asp + Glu) was 14, and the total number of positively charged residues (Arg + Lys) was 32. The instability index was calculated to be 44.87, and the adipose index was 109.06. The minimum value of the strongest hydrophilic amino acid was − 2.089, and the maximum value of the strongest hydrophobic amino acid was 3.156. The average hydrophilic coefficient was 0.530, suggesting that this is a hydrophobic protein (Fig. 3). SignalP was used to predict the signal peptide position of the protein, and the average D value was 0.416, which is less than the threshold 0.450. Thus, the existence of signal peptide is not likely (Fig. 4). Using online analysis of SOPMA server, the secondary structure of GHITM protein was predicted to be: 161 alpha helices (Hh), accounting for 47.08%; 59 beta folds (Ee), 17.25%; 21 beta angles (Tt), accounting for 6.14%; There were 101 crimps without regulation (Cc), accounting for 29.53% (Fig. 5).

The subcellular localization of the GHITM protein was predicted using the software PSORT II Prediction. In total, 55.6, 33.3 and 11.1% of the GHITM protein was distributed in the endoplasmic reticulum, plasma membrane and vacuole, respectively. Therefore, the GHITM protein appears to be mainly distributed in cytoplasm.

**Fig. 6:**
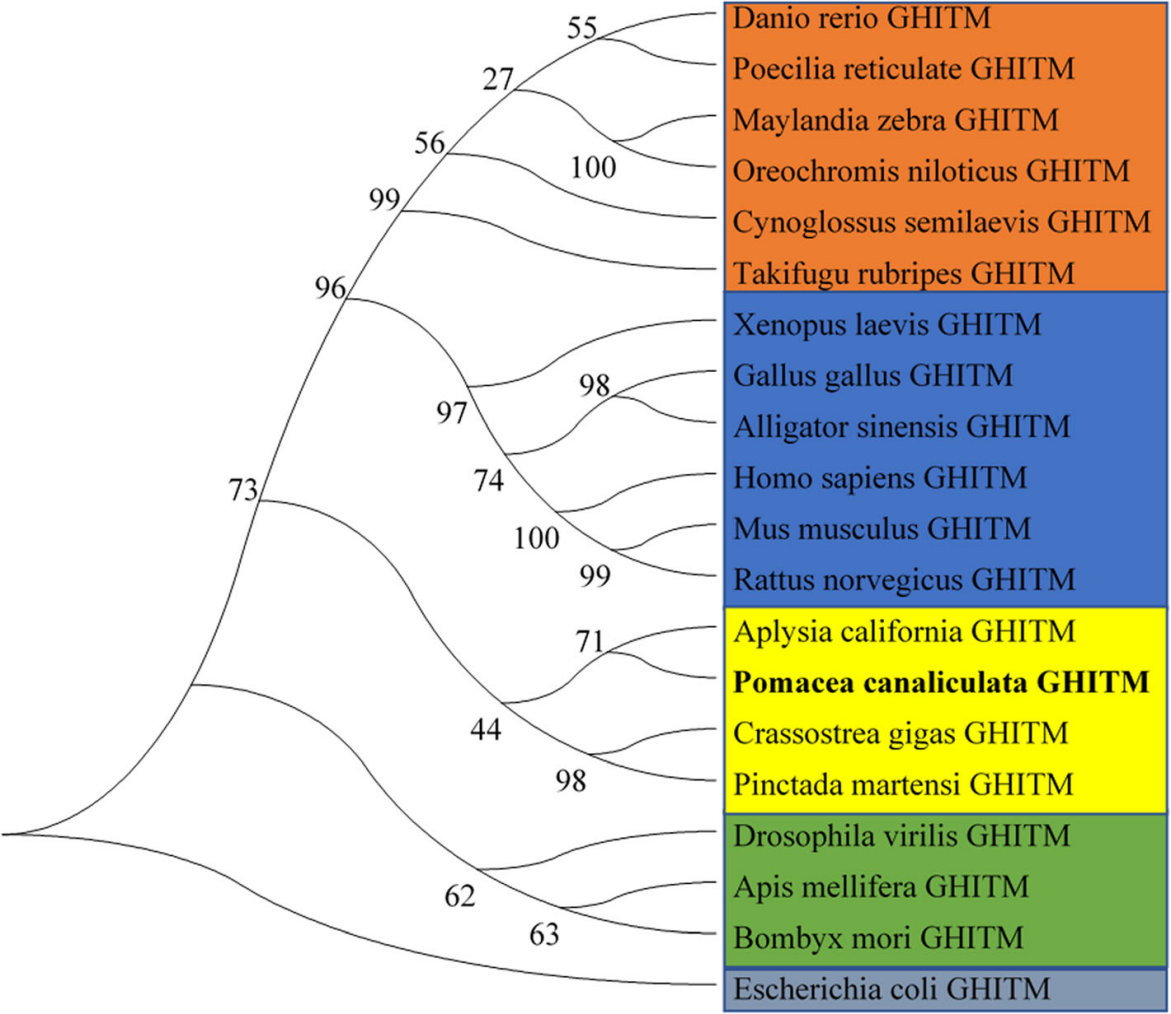
The phylogenetic tree of GHITM amino acid sequences from 20 species

A phylogenetic tree was constructed using the MEGA 7.0 program with the Neighbor-Joining method according to the deduced amino acid sequences of GHITM and other previously reported GHITM proteins. The phylogenetic tree revealed that these GHITMs were divided into three clusters, vertebrates, mollusks, and insects. The GHITM of golden apple snails was grouped into the clade containing GHITMs from molluscs. Moreover, it was most closely related to that of *Aplysia california* (Fig. 6), and they are both Gastropods.

### Expression level of GHITM in different tissues and different growth phases of the Golden apple snail

**Fig. 7:**
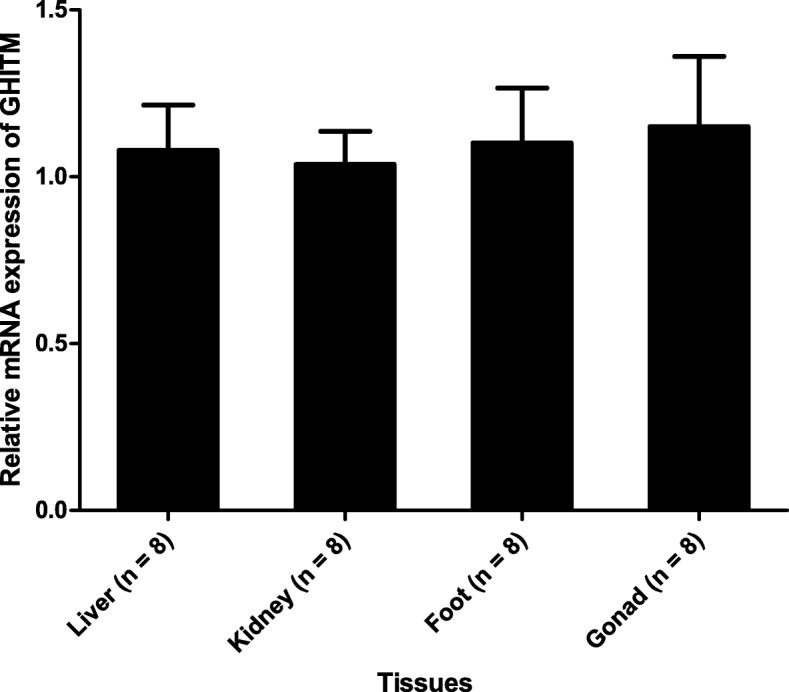
Expression analysis of GHITM in different tissues of the golden apple snail (*n* = 8). Each bar represents the mean of 8 independent experiments performed in duplicate. The real-time quantitative PCR (qPCR) data were expressed as the mean ± SE

**Fig. 8:**
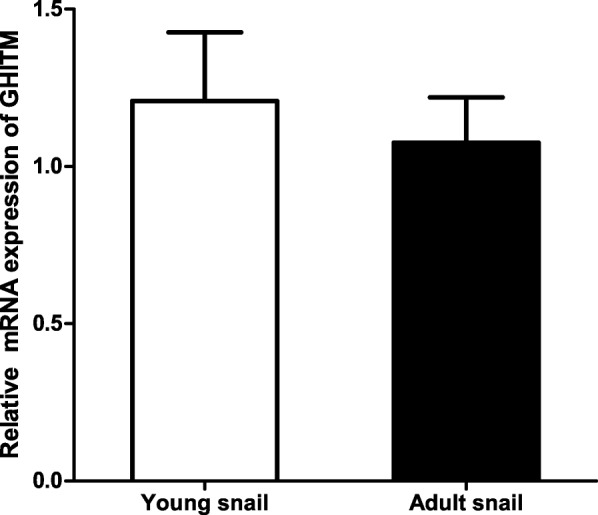
GHITM mRNA expression during different growth phases of the golden apple snail (n = 8). Each bar represents the mean of 8 independent experiments performed in duplicate. The real-time quantitative PCR (qPCR) data were expressed as the mean ± SE

To explore the expression pattern of the GHITM gene in different tissues, qPCR was performed using total RNA from liver, kidney, gonad and foot. GHITM gene is generally expressed in various tissues without significant difference (Fig. 7). In addition, to detect the expression pattern of the GHITM gene during different growth phases, RT-qPCR was performed with total RNA from young (6-months old) and adult (2-years old) golden apple snails. The GHITM transcript levels were not significantly different between 6-month-old and 2-year-old golden apple snails (Fig. 8).

### The siRNA-mediated Down-regulation of target genes

**Fig. 9:**
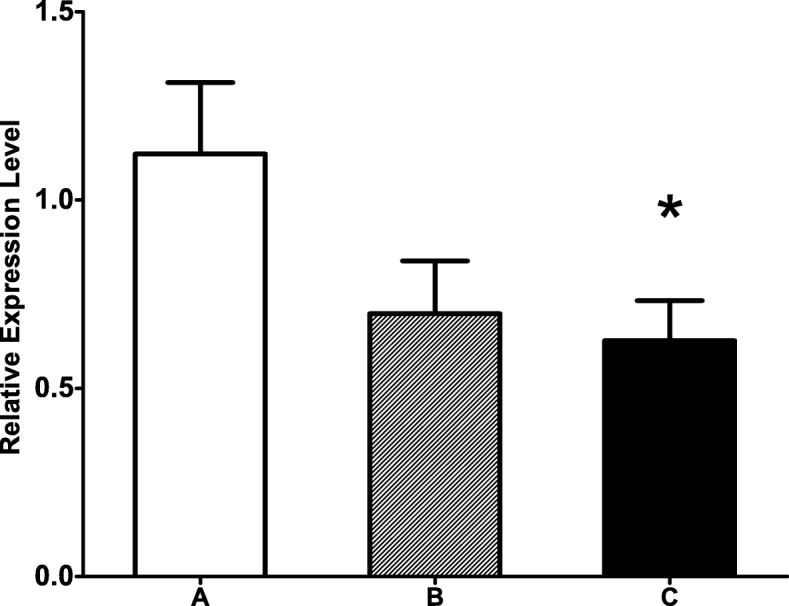
Expression of GHITM gene in foot after RNAi, A was a control group (*n* = 10), B and C were two experimental groups (*n* = 8). Each bar represents the mean of 8 independent experiments performed in duplicate. The (*) indicates statistically significant difference (*P* < 0.05). The real-time quantitative PCR (qPCR) data were expressed as the mean ± SE

To reveal the function of the GHITM gene, RNAi was performed on healthy golden apple snails. Because the foot had the highest GHITM expression level of the tested organs, it was used to determine changes after RNAi. The expression of the GHITM gene was down-regulated by RNAi, with a significantly lower expression level being detected when 10 μg siRNA + 50 μl PBS was used (Fig. 9).

### Apoptosis index measurement

**Fig. 10:**
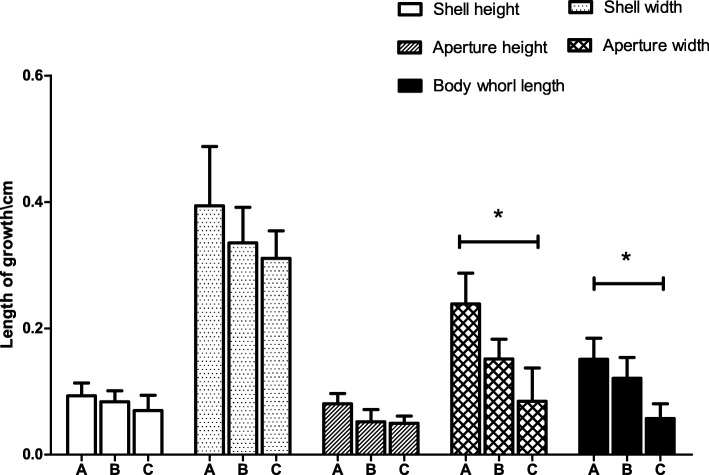
The activity of Caspase-3 in foot of the 3 groups golden apple snails (*n* = 8). Each bar represents the mean of 8 independent experiments performed in duplicate. The double-asterisk (**) indicates statistically significant difference (*P* < 0.01). The data were expressed as the mean ± SE

The activity levels of caspase-3 in the feet of the three groups were measured after the RNAi experiment. The activity levels of caspase-3 in the two experimental groups were significantly higher than that of the control group (Fig. 10).

### The effects of RNAi on the growth rate

**Fig. 11:**
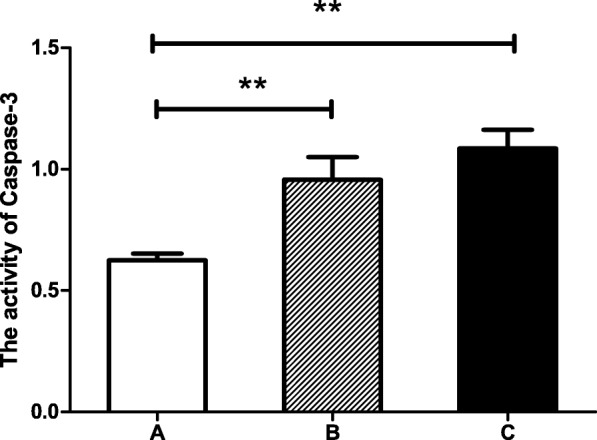
The change of shell height, shell width, aperture height, aperture width and body whorl length (A is control group, B and C are experimental groups, *n* = 8. Each bar represents the mean of 8 independent experiments performed in duplicate. The (*) indicates statistically significant difference)

The changes in the growth indexes, including shell height, shell width, aperture height, aperture width and body whorl length, of the control and experimental groups were calculated. The calculation method subtracted the first measurement value from the second measurement value. The change in the growth of the control group was greater than those of the two experimental groups, and the aperture width and body whorl length of the control group were significantly greater than those of the C group (Fig. 11).

## Discussion

Since the first discovery of GHITM in mice in 2001, it has been identified successively in vertebrates and invertebrates, such as human, chicken, zebrafish and oyster [23]. In this study, the GHITM gene was identified for the first time in golden apple snail and this is also the first report of full-length cDNA cloning and sequence analysis of GHITM from the golden apple snail.

Bioinformatics methods, including computer simulations and predictions of gene structure and biological function, have been widely used in pathogen detection, gene information analysis, vaccine development and other fields [31]. In this study, the GHITM gene was cloned and its physical and chemical properties, hydrophilic and hydrophobic properties, signal peptide prediction, subcellular localization prediction and other bioinformatics analyses were also performed to predict the basic structure, properties and distribution of the GHITM gene of the golden apple snail. In addition, a phylogenetic tree was constructed using the GHITM amino acid sequences of 20 species, and it revealed that the GHITM sequence of the golden apple snail was highly homologous with that of *A. california*, which was consistent with the classification provided by biological evolution. These results provide information for the further study of the gene’s function.

The transcript levels of the GHITM gene in different tissues (liver, kidney, gonad and foot) and different growth phases (6-months old and 2-years old) were assessed using fluorescent qPCR. The results showed that GHITM gene was expressed in different tissues and growth stages, and also widely throughout individuals, which was basically consistent with previous studies on other species [23, 32]. To some extent, it was speculated that the gene may play a role in different tissues and different growth phases of golden apple snail.

**Fig. 12:**
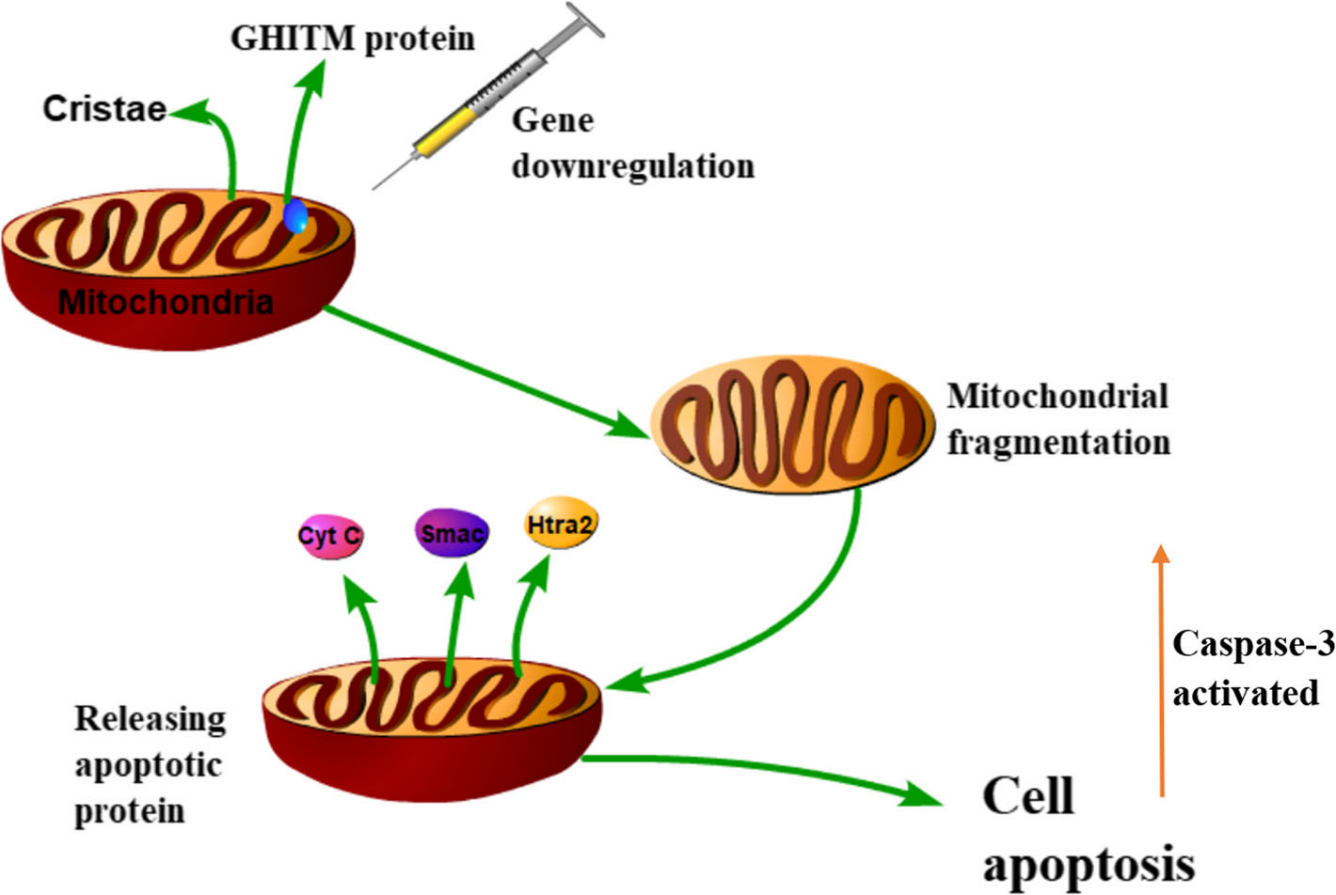
Predictive pathway map of apoptosis induced by downregulation of GHITM gene

Mitochondria play an important role in the early stage of apoptosis. The release of pro-apoptotic proteins in mitochondria activate downstream caspase proteins and leads to apoptosis [33]. Caspase-3 belongs to the CED (*Caenorhabditis elegans* death gene)-3 subfamily of the Caspase family [34]. In a normal state, caspase-3 exists in the cytoplasm in the form of zymogen with no activity. However, caspase-3 was activated when apoptosis occurs. We reduced the expression of the GHITM gene in the experimental group by RNAi technology, and then tested the activity of caspase-3 in the control group and the experimental group. The activity level of caspase-3 in the two experimental groups were significantly higher than that in the control group, indicating that apoptosis of the two experimental groups was severe. Therefore, it was speculated that the GHITM gene was involved in inhibiting apoptosis in golden apple snails. GHITM was reported to be a mitochondrial protein that functions to maintain cristae organization [33, 35]. We presumed that down-regulation of the GHITM gene leads to mitochondrial rupture, which leads to the release of apoptotic factors such as cytochrome c, Smac (Second mitochondria-derived activator of caspase), and Htra2 (HtrA serine peptidase 2) [36], and ultimately induces apoptosis and activates Caspse-3 (Fig. 12).

The effect of the GHITM gene on the growth of the golden apple snail was verified by comparing the changes in several growth indexes between the RNAi experiment groups and the control group after 20 d. Therefore, it was deduced that the growth rates of the golden apple snails in the experimental groups were slow owing to the RNAi-based disrupted expression of the GHITM gene. Thus, the GHITM gene could affect the growth of the golden apple snail. The golden apple snail was introduced into Asia as a human food, but it has become a serious pest of important agricultural crops as well as a threat to aquatic and wetland ecosystems [37]. Currently, the prevention and control of the golden apple snail are concentrated on mechanical methods, biological control and the use of chemical pesticides [38, 39]. The study results provide a theoretical basis and new insights into the prevention and control of this agricultural pest.

As a biological process, apoptosis is carried out during maturation, remodeling, growth, developmental, defense and immunity processes [40, 41]. There were some studies on inhibiting apoptosis and promoting cell growth, for example, transcriptional co-activators with PDZ-binding motif (TAZ) can promote cell growth and inhibits Celastrol-induced cell apoptosis [42], HSP90 (Heat Shock Protein 90) can inhibit apoptosis and promote growth by regulating HIF-1α (Hypoxia Inducible Factor-1α) abundance in hepatocellular carcinoma [43]. In this study, it was verified that the GHITM gene played roles in inhibiting apoptosis and promoting growth in the golden apple snail, which could increase our understanding of apoptosis and growth mechanisms in molluscs.

## Conclusions

These results demonstrated that the GHITM gene was involved in apoptosis and growth in golden apple snail, which provides a theoretical basis and new insights into the prevention and control of the growth and reproduction of the golden apple snail as an agricultural pest.

## Methods

### Animals

Healthy golden apple snails (Adult snail body weight = 25 - 28 g, young snail body weigh = 8 - 10 g) were collected from the Xiamen, Fujian Province, China, and maintained in freshwater in an incubator at 25 ± 2 °C (12-h:12-h light: dark photocycle) before the experiment. The golden apple snails were fed Chinese cabbage daily.

### RNA extraction and cDNA synthesis

Total RNA was extracted from the golden apple snails using the TRIzol (TaKaRa Biotechnology, Dalian, China) method. Briefly, samples were dissociated in TRIzol, then extracted using chloroform to eliminate proteins, precipitated with isopropyl alcohol and successively washed with 75% ethanol. RNase inhibitor was added into the extracted RNA to ethanol. RNase inhibitor was added into the extracted RNA to eliminate RNase. The quality, purity and integrity of the RNA were determined using a spectrophotometer (A 260/A 280) and agarose gel electrophoresis. The full-length cDNA was synthesized using SMARTer® RACE 5′/3′ Kit following the instructions in the User Manual (634,858, TaKaRa Biotechnology, Dalian, China). The RNA and cDNA were stored at − 80 °C for further analyses.

### GHITM cDNA cloning

A partial sequence (GBZZ01005442.1) was predicted to be the GHITM cDNA of the golden apple snail by a comparison between the transcriptome (GBZZ00000000.1) and GenBank database. Specific primers were designed based on the predicted sequences (Table 1). PCR was conducted at 95 °C for 3 min followed by 35 cycles of 95 °C for 30 s, 55 °C for 30 s and 72 °C for 30 s. A final extension step at 72 °C for 5 min was performed, and the PCR products were cloned into the pMD-18 T vector (6011, TaKaRa Biotechnology, Dalian, China) and sequenced. Based on the sequenced cDNA fragment, the 5′- and 3′-ends of the GHITM fragment were cloned using the SMARTer® RACE 5′/3′ Kit (634,858, TaKaRa Biotechnology, Dalian, China).

**Table 1:** Primers used in gene cloning and RT-qPCR

Primer	Sequences (5′-3′)	Purpose
GHITM-F	ACTTCCTTTTGCTGCTTCC	Conventional PCR
GHITM-R	GGAGCACTTGTCTACTATGGGT	
5′- GHITM-R	GATTACGCCAAGCTTCCTGTCTTTGCTCAACGCCCTTAT	RACE
3′- GHITM-F	GATTACGCCAAGCTTCCTGCGCTTAACTTCTTCTGACCAC	
Long primer	CTAATACGACTCACTATAGGGCAAGCAGTGGTATCAACGCAGAGT	
Short primer	CTAATACGACTCACTATAGGGC	
qGHITM-F	CGCTGTTTCTCCCTACCAT	qRT-PCR
qGHITM-R	AAGCCAGGAATTTCTCATC	
q18S RNA-F	AATACATGCAAACCAGCTCC	
q18S RNA-R	ATTTTTCGTCACTACCTCCC	

### Bioinformatics analysis of GHITM

The nucleic acid sequence was transformed into the amino acid sequence using Editseq of DNAStar (Madison, Wisconsin, USA). The amino acid isoelectric point and molecular mass of the protein were analyzed by ProtParam (http://web.expasy.org/protparam/). A hydrophilic and hydrophobic profile of the GHITM gene was predicted using the software Protscale (https://web.expasy.org/protscale/). The SignalP 4.1 server (http:/www.cbs.dtu.dkP/ services/SignalP-4.1/) was used to predict the signal peptide of GHITM. The subcellular localization and protein function of the GHITM protein were analyzed using PSORT II Prediction (http: r/p sort.hgc. jp/form2.html) and Protfun2.2 (http://www.cbs.dtu.dk/services/prot function/). The secondary structure of GHITM protein was predicted Using online analysis of SOPMA server (https://omictools.com/sopma-tool). A multiple sequence alignment of GHITM with other GHITM protein sequences from mollusk, crustaceans and vertebrates was performed using the ClustalW Multiple Alignment, and a phylogenetic tree was constructed with the Neighbor-Joining method using MEGA 7.0 (Center for Evolutionary Medicine and Informatics, The Biodesign Institute).

### GHITM mRNA expression in different tissues and different growth phases

The transcript levels of the GHITM gene in different tissues (liver, kidney, gonad and foot) and various growth phases (6 months and 2 years) were assessed using fluorescent quantitative PCR (qPCR) with the SYBR@ Premix Ex Taq™ (DRR081A, TaKaRa Biotechnology, Dalian, China). A pair of specific primers qGHITM -F/R (Table 1) for qPCR was designed, and the q18S RNA gene was used as an internal control. The qPCR profile was 95 °C for 2 min followed by 39 cycles at 95 °C for 5 s and 60 °C for 30 s. The amplification efficiency (E) of each primer pair was calculated when the curve of the Ct [Log (cDNA dilution)] was generated. The E value of qGHITM-F/q18S RNA-R was 100.6%. The relative transcription levels of the *GHITM* gene from different samples (*n* = 8) were analyzed using the 2^−ΔΔCt^ method [44].

### The growth index measurement

A total of 30 golden apple snails were randomly divided into 3 groups. A control group (named A) and two experimental groups (named B and C). The growth indexes, including shell height, shell width, aperture height, aperture width and body whorl length, were measured [45].

### RNAi experiment

Based on the sequence of GHITM, the target siRNA was designed and synthesized by Sangon Biotech (Shanghai). The RNA interference location for GHITM was labeled as 271TP. The following siRNA sequences were used in this experiment: Sense: GCUCUACGGCCUUGACAUUTT and Antisense: AAUGUCAAGGCCGUAGAGCTT.

The siRNAs of the GHITM gene were dissolved in PBS buffer for foot muscle injection. The experimental groups (B and C) were injected with the following treatments: 5 μg siRNA + 50 μl PBS and 10 μg siRNA + 50 μl PBS, respectively, while the control group (A) was only injected with 50 μl of PBS. Preliminary experiments showed that the effects of a PBS injection were the same as no injection; consequently, the PBS injection group was selected as the control group. The foot muscle injection was conducted once every 4 d, for 20 d.

### Verification of RNAi interference effects

After 20 d of siRNA injection, members of the three groups living golden apple snails (*n* = 8) were sacrificed, and their foot tissues were sampled. Total RNAs were extracted from foot tissues and reverse transcribed into cDNA for real-time qPCR experiments. The mRNA expression levels were assessed using the SYBR@ Premix Ex Taq™ (DRR081A, TaKaRa Biotechnology, Dalian, China) and analyzed using the 2^−ΔΔCt^ method.

### Apoptosis index measurement

To verify the possible role of GHITM in apoptosis, the activity levels of caspase-3 in the feet of the three groups of golden apple snails (*n* = 8) were also measured after the RNAi experiment. After homogenization of the tissue, Ac-DEVD-pNA was added. After the sample turned yellow, the absorbance of A400nm was measured by a microplate reader, and the relative caspase 3 activity was calculated according to the ratio of the absorbance of the apoptosis-induced cells to the absorbance of the blank control cells (Caspase 3 Activity Assay Kit, G015–1-3, Nanjing Jiancheng Bioengineering Institute, Nanjing, China). Further reflects the degree of apoptosis of tissue samples.

### The growth index measurement after RNAi experiment

To determine the possible function of GHITM in maintaining normal growth, we measured the growth index and observed the changes between experimental groups and the control group (*n* = 8) after the RNAi experiment.

## Data Availability

The datasets used and/or analyzed during the current study are available from the corresponding author on reasonable request. The cDNA sequences generated during the current study are available in the [GenBank] repository, [MN219409].

## Abbreviations

BI: BAX inhibitor
Caspase: Cysteine-requiring Aspartate Protease
CED: *Caenorhabditis elegans* death gene
GHITM: Growth hormone inducible transmembrane protein
HIF-1α: Hypoxia Inducible Factor-1α
HSP90: Heat Shock Protein 90
Htra2: HtrA serine peptidase 2
qPCR: Real-time quantitative PCR
RACE: Rapid amplification of cDNA ends
RNAi: RNA interference
Smac: Second mitochondria-derived activator of caspase
TAZ: Transcriptional co-activators with PDZ-binding motif
UTR: Untranslated Region
